# Giant deep lobe parotid tumor removal via total parotidectomy without mandibulotomy. A simple and safe technique

**DOI:** 10.4317/jced.57202

**Published:** 2021-03-01

**Authors:** Jordi Borrás-Ferreres, Miguel Armengot-Carceller

**Affiliations:** 1 DDS, MS. Professor of the Master Degree Program in Oral Surgery and Orofacial Implantology (EFHRE International University); 2 MD, PhD. Head of the Department of Otorhinolaryngology, La Fe University and Polytechnic Hospital, Valencia, Spain. Professor of the Department of Surgery, Faculty of Medicine, Valencia University, Valencia, Spain

## Abstract

The transmandibular route is often combined with the transparotid-transcervical approach when extensive surgical field exposure is required, as in the case of deep parotid lobe tumors measuring over 4 cm in size. This procedure implies great morbidity and prolongs surgery time. Furthermore, in cases where additional lip division is performed, the aesthetic outcomes may be poorer. A description is made of the technique used for the removal of giant pleomorphic adenomas of the parapharyngeal space, without mandibulotomy.

** Key words:**Parapharyngeal space tumor, pleomorphic adenoma, transcervical-transparotid approach, transmandibular approach.

## Introduction

Salivary gland tumors represent less than 4% of all head and neck tumors (1,2). Pleomorphic adenoma (PA) accounts for about 65% of all salivary gland tumors, and most of them affect the major salivary glands – particularly the parotid gland (2,3). The majority originate in the superficial lobe, but the deep lobe is affected in 10-20% of the cases, with tumor growth medial and occupation of the parapharyngeal space (PPS) (1,4). Because of their slow growth (5,6) and the absence of symptoms (7), these tumors are typically diagnosed late, when the lesion measures over 2.5-3 cm in size, and manifests as a mass causing medial displacement of the lateral oropharyngeal wall (5,8). In the case of large PAs, planning of the surgical approach requires the decision of whether or not to perform mandibulotomy.

We present two cases of giants PAs of the deep lobe of the parotid gland subjected to total parotidectomy without mandibulotomy thanks to digital displacement of the tumors from the oropharynx. This resulted in lesser patient morbidity and shorter hospital stay.

## Case Report

Two women aged 49 and 31 years presented with similar clinical features: pharyngeal foreign body sensation, dysphagia without pain on swallowing, lack of voice resonance and nocturnal snoring. The explorations revealed a rounded firm protrusion, covered by normal mucosa, located in the left tonsillar region and partially occupying the oropharynx. Palpation revealed no adenopathies.

**Figure 1:**
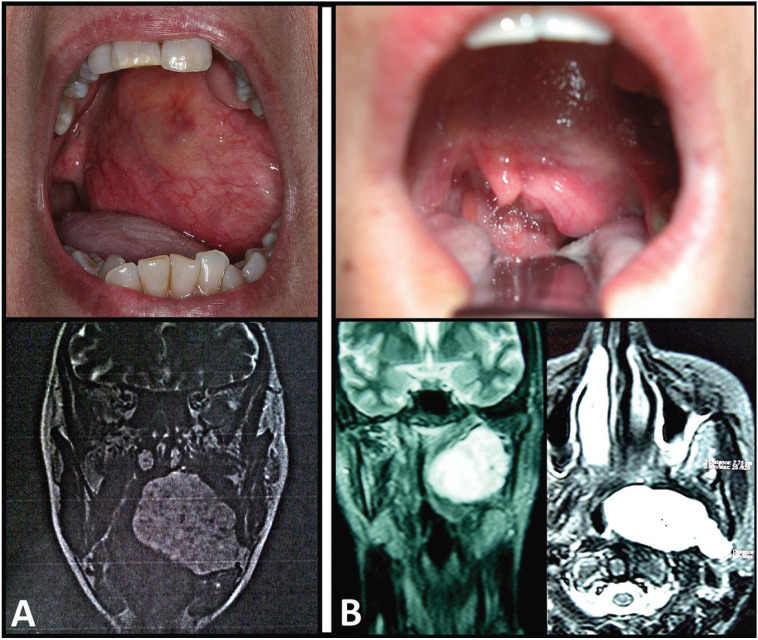
Preoperative clinical view of both patients. A. Intraoral and coronal MRI views of case 1. The pleomorphic adenoma located in the left parapharyngeal space encompasses the entire left soft palate. The tongue is constricted and the uvula is displaced to the right. B. Intraoral, coronal and axial MRI views of case 2.

The tumor origin was established by magnetic resonance imaging (MRI) in both cases. The lesion measured over 60 mm in maximum diameter, and was well delimited, lobulated and encapsulated. It was seen to be in direct contact with the deep lobe of the left parotid gland, and extended towards the pre-stylohyoid compartment of the PPS through the stylomandibular tunnel. In both cases the lesion was seen to be hypointense in T1- and hyperintense in T2-weighted sequencing. The tumor exerted a mass effect and displaced and compressed the airway lumen to the right, distorting the morphology of the rhinopharyngeal cavum in its cephalad growth, and the oropharynx in its caudad extension (Fig. 1).

Fine needle aspiration biopsy (FNAB) via the transoral route indicated PA in both patients.

**Figure 2:**
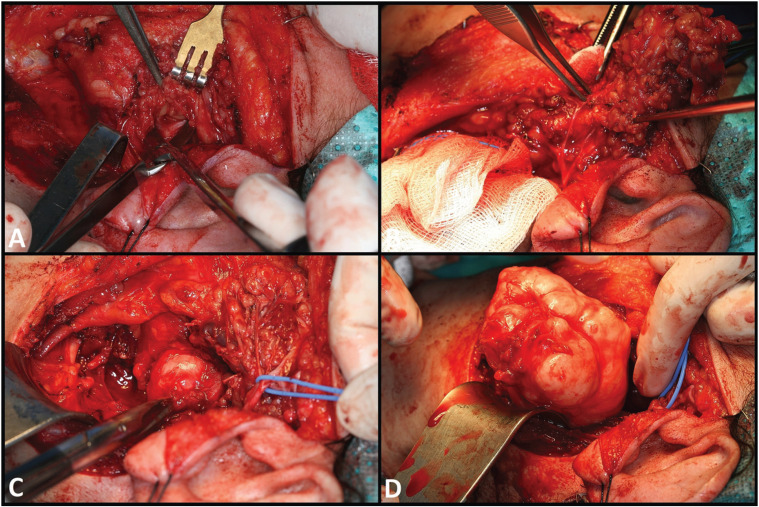
Surgery in case 1. A. Location of the trunk of the facial nerve. B. Removal of the superficial lobe of the parotid gland. C. Exposure of the pleomorphic adenoma following resection of the deep lobe of the parotid gland. D. Tumor resection is carried out with the application of digital pressure from the oropharynx.

Surgical removals under general anesthesia were indicated, with total parotidectomy and preservation of the facial nerve. A transparotid-transcervical approach was used, with monitoring of the facial nerve. A skin incision was made from the upper preauricular region, from the upper edge of the tragus cartilage, descending vertically immediately anterior to the tragus. On reaching the point just below the earlobe, incision was directed towards the reference corresponding to the tip of the mastoid process and a curve was then described downwards and anteriorly towards the hyoid bone. A skin flap was raised to fully expose the gland, with identification of the external jugular vein and auriculotemporal nerve. Then, an incision was made in the zone of the medial portion of the sternocleidomastoid muscle to visualize the posterior belly of the digastric muscle as reference for localization of the facial nerve. The parotid gland was detached from the cartilage of the external auditory canal (EAC), and the trunk of the facial nerve was identified in the depth of this zone following sectioning of the temporoparotid fascia (Fig. 2A). We then detached the superficial lobe of the parotid from the branches of the facial nerve, reflecting the gland tissue anteriorly (Fig. 2B). Following resection of the superficial lobe of the gland, the extracranial portion of the facial nerve lay exposed over its entire trajectory, with the deep lobe of the parotid positioned in depth to the nerve. Downward traction upon the lobe allowed us to detach it from the cervicofacial branch of the nerve. Following exeresis, the external carotid artery was seen in depth, together with the tumor (Fig. 2C). Although the lesion was not completely exposed, its dissection and extraction were facilitated by displacing it outwards applying digital pressure from the oropharynx. This maneuver avoided the need for osteotomy of the ascending ramus of the mandible to secure full visualization (Fig. 2D).

**Figure 3:**
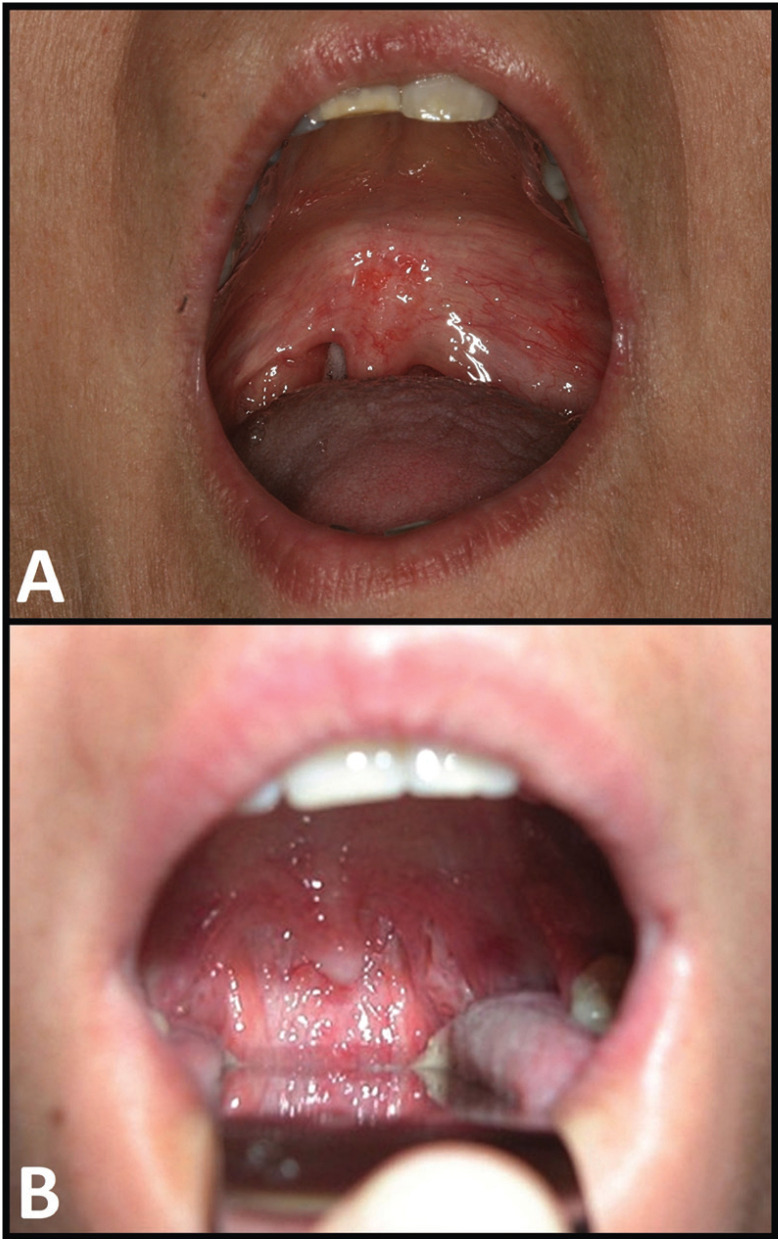
Postoperative clinical view of both patients. A. Case 1. B. Case 2.

The postoperative courses proved uneventful, and no relapse were observed 5 and 7 years after tumors removals (Fig. 3).

## Discussion

PAs can manifest at any age, but are most common between the fourth and fifth decades of life, and are more prevalent in women than in men (3,9). These are the most frequent tumors of the parotid gland, representing 60.3% of all parotid tumors according to Kizil *et al.* in their study of 292 parotid gland tumors (3). Although PAs are benign, recurrences are observed in about 5% of all cases after surgery (10,11). Recurrence is mainly observed in those cases characterized by focal absence of the capsule that often envelops the tumors, with microscopic tumor projections into the surrounding parotid tissue (12), or in cases of capsule rupture occurring during surgical removal of the tumor (10,13). Simple enucleation of the tumor may leave these projections behind and thus increase the risk of recurrence (11,12). Removal of the affected lobe is therefore necessary, as evidenced by Ayoub *et al.* in a series of 182 cases in which the recurrence rate was only 1.7% (12).

Tumors affecting the PPS are rare and represent only 0.5% of all head and neck tumors (4,5,10) – with approximately 80% of the lesions being benign (6,8,14). Salivary gland tumors are the most frequent lesions, with PA originating in the deep lobe of the parotid gland being the predominant tumor (4,5,7). The management of these tumors is a challenge for surgeons, due to the presence of vital neurovascular elements (5,15). PAs of the deep lobe are generally located in the anterior compartment of the PPS, representing 50% of the benign lesions in that location, followed by neurogenic tumors (5,6). In contrast, the latter are the most common tumors in the posterior compartment (7).

PAs of the deep lobe of the parotid gland are generally diagnosed as an asymptomatic mass located in the oropharynx. Because of the slow growth of these lesions, the diagnosis is established late, when the tumor generally exceeds 3 cm in size (3,6). These data are consistent with the observations of Kato *et al.*, who analyzed the mean size of 10 PAs of the PPS and found the diameters to range between 35-57.3 mm (9). In addition to the mass effect, the clinical manifestations include a foreign body sensation in the throat, swallowing difficulties and voice alterations (as in our patients), as well as trismus, otological symptoms secondary to Eustachian tube obstruction and, more rarely, obstructive sleep apnea (1,4,8) (as also occurred in our patients). The appearance of facial pain or paralysis of the cranial nerves is associated to malignant degeneration (4).

The diagnosis requires an imaging study to fully determine the characteristics of the lesion. Both computed tomography (CT) and MRI can be used for this purpose (7,9). However, MRI is the preferred technique, since it offers useful information about the location of the PA and its extent, and is able to distinguish between deep lobe tumors and neurogenic tumors, paragangliomas or tumors of the carotid body (5). Furthermore, MRI allows better definition of the soft tissues and their relation to the internal carotid artery and adjacent structures (1,5). On the other hand, CT is able to detect bone invasion by malignant tumors (9). It is very important to distinguish between PAs arising from the deep lobe of the parotid gland and those originating in ectopic salivary glands of the PPS, since this can condition the surgical approach. The identification of parapharyngeal adipose tissue between the deep lobe of the parotid gland and the tumor in the MRI and CT studies is indicative of an extra-parotid lesion (6).

Although a transoral biopsy under local anesthesia can be performed to obtain a preoperative histopathological diagnosis, it has been reported that this practice should be avoided, since opening of the capsule enveloping the tumor increases the risk of recurrence (10,13) and of bleeding in vascular tumors (4,5,15). Accordingly, transoral FNAB is regarded as the diagnostic technique of choice (1,15).

Many surgical techniques for the management of tumors of the PPS have been described, fundamentally in reference to PAs. The main strategies involve the transcervical, transoral, transparotid-transcervical and transmandibular approaches (6,8,10).

The transoral approach is generally reserved for small benign extra-parotid tumors measuring under 2 cm in size and originating in the anterior compartment (8,10), though some authors such as Hussain *et al.* use it for PAs originating in the parotid gland, defending the advantages of minimally invasive surgery (14).

The transcervical approach is reserved for benign extra-parotid tumors such as PAs originating in the minor salivary glands of the PPS and measuring under 4 cm in size (8,10).

In contrast, the transparotid-transcervical approach is the most widely used strategy, and is reserved for PAs of the deep lobe of the parotid gland measuring under 4 cm in size (10,16). This approach involves total parotidectomy with preservation of the facial nerve, combined with a cervical access to dissect the cranial nerves and vascular structures - thus allowing safe removal of the tumor together with the deep lobe of the parotid gland (8). In addition, this approach offers good visualization, bleeding control and the identification of vessels and nerves, and allows us to complete lymph node removal in the event of malignant lesions (8,10). However, the transparotid-transcervical approach is associated to complications such as facial nerve damage, facial deformities, antiesthetic sequelae secondary to scarring, or the appearance of Frey syndrome (2,6,14). In order to avoid these complications, some authors such as Sesenna *et al.* propose preservation of the superficial lobe of the gland during the surgical approach, and affirm that this measure does not increase the recurrence rate (16). Nevertheless, the duration of patient follow-up was 6 years on average; their conclusions therefore must be viewed with caution. Also, the exposure and identification of the vascular and nervous structures is facilitated with the excision of the superficial lobe, preventing its injury. Consequently, it facilitates the excision of deep lobe tumors without mandibulotomy (15). Other investigators such as Infante *et al.* combine the transparotid approach – preserving the superficial lobe of the gland – with a transoral approach to remove giant PAs of the deep lobe (6). However, it must be noted that these authors recorded a high incidence of tumor capsule rupture.

Although the transcervical and transcervical-transparotid approaches are well known procedures in PPS surgery, they are rarely recommended for large tumors. On the other hand, a mandibular osteotomy has been advocated in the literature when a malignant tumor, a tumor located high within the PPS, or a tumor over 4 cm in size needs to be resected (1,10). Its anatomically complex conFiguration is a great limitation for surgeons when approaching the PPS, and its exposure with the mandible visible is very poor. This sometimes requires blunt dissection of the tumor without good exposure, and theoretically may cause damage to the nervous and vascular structures lying behind the mass. The transmandibular approach offers wider access, thus reducing the intraoperative risks, but requires one or more osteotomies and therefore takes longer and is technically more demanding (10). Despite better PPS exposure, mandibulotomy increases patient morbidity and prolongs hospital stay.

Although our tumors were over 6 cm in size, they were successfully removed adopting a transparotid-transcervical approach combined with digital pressure applied from the oropharynx - this being a key surgical maneuver in removal of the tumor. We believe that in expert hands, large tumors can also be removed using these approaches without mandibulotomy, reducing patient morbidity, affording better aesthetic and functional outcomes and without additional surgical risks.

## Conclusions

Giant pleomorphic adenoma of the deep lobe of the parotid gland located within the parapharyngeal space can be successfully removed through a transparotid-transcervical approach without the need for mandibulotomy or oropharyngeal incisions. The approach is similar to that of any parotidectomy for tumor removal, though dissection of the deep lobe with the tumor involves the application of digital pressure from the oropharynx without damaging the mucosa. This approach affords benefits for the patient by shortening surgery time and hospital stay, while also facilitating the radical management required by tumors of this kind.
